# Morphology and head morphometric characters of sperm in Thai native crossbred stallions

**DOI:** 10.1186/1751-0147-50-41

**Published:** 2008-10-22

**Authors:** Kanittha Phetudomsinsuk, Kaitkanoke Sirinarumitr, Aree Laikul, Anuchai Pinyopummin

**Affiliations:** 1 Faculty of Veterinary Medicine, Kasetsart University, Kamphaeng Saen Campus, Nakhon Pathom 73140, Thailand; 2 Center for Agricultural Biotechnology, Kasetsart University, Kamphaeng Saen Campus, Nakhon Pathom 73140, Thailand

## Abstract

**Background:**

One of the semen quality parameters use to determine fertility is the percentage of sperm that express normal morphology. Sperm head morphometry is also correlated with fertility. The objectives of this study were 1) to investigate the sperm morphology and normal sperm head morphometry of Thai native crossbred stallions, and 2) to compare our results with the characteristics of proven fertile sperm from purebred stallions.

**Methods:**

Semen samples were collected monthly from nine stallions, of which five were Thai native crossbred (T) and four were purebred of proven fertility (F: F1 was a Standard-bred; F2 was a Warm-blood; F3 and F4 were Thoroughbreds). All the animals were aged between 5 and 12 years. Sperm morphological examination was performed using formaldehyde-fixed samples under phase-contrast microscopy (1000×). Normal sperm head morphometry characteristics were measured by Computer-Assisted Semen Analysis (Hamilton Thorne, USA.) after applying the Harris' haematoxylin staining technique.

**Results:**

The percentages of morphologically normal and abnormal sperm varied among individual stallions in both the T and F groups. The mean percentage of morphologically normal sperm was not significantly different (P > 0.05) between T and F stallions (mean ± SE, 49.7 ± 1.3 and 48.1 ± 2.8, respectively). A comparison between the T and F sperm heads revealed that all the dimensional parameters were significantly different (P < 0.05). The coefficients of within-animal variation (CVs) ranged from 2.6 (shape factor 1) to 7.5 (elongation) and 2.9 (shape factor 1) to 8.1 (elongation) in T and F, respectively. In the case of the T group, those sperm head parameters that featured a low within-animal CV and a high between-animal CV were perimeter (2.9, 19.1), shape factor 1 (2.6, 25.8) and shape factor 3 (3.8, 32.0). In the case of the F group, only shape factor 1 (2.9, 26.1) featured such characteristics.

**Conclusion:**

We found variability in the percentage of morphologically normal and abnormal sperm, as well as in sperm head dimensions among Thai native crossbred stallions, and these results were similar to those of purebred stallions. Our findings demonstrate that the heads of the T sperm specimens were larger and rounder than that of the F sperm. Perimeter, shape factor 1 and shape factor 3 could be used as parameters for the identification of individual T stallions based on a sperm sample.

## Background

Recent studies have shown that male fertility does not only depend on the absolute number of viable, motile, morphologically normal sperm that can be inseminated in a female. Rather, a more important parameter appears to be the functional competence of sperm cells – since this cannot be evaluated using a single variable, researchers have proposed that semen samples should be subjected to multi-parametric analysis [1-3]. However, gross morphological classification of the sperm in order to assess male fertility can be used as a practical screening tool and is already a part of the breeding soundness examination (BSE) that is used in Thailand for all domestic species, including horses [4]. An ejaculation containing a minimum of one billion morphologically normal, progressively motile sperm specimens in each of two ejaculates sampled at any time during the year is the guideline for satisfactory stallion BSE as codified by the Society for Theriogenology [4,5]. Under light microscopy, a significant increase in the morphological abnormality of sperm samples was observed in stallions that were either infertile or of dubious fertility [6,7]. The average stallion had approximately 50% morphologically normal sperm, but some stallions with less than 40% morphologically normal specimens may achieve acceptable pregnancy rates if a minimum threshold number of normal sperm are present [8].

Sperm head morphometry assessed by Computer-Assisted Semen Analysis (CASA) has been shown to correlate with fertility in various species including horses [9], boar [10,11], Iberian red deer [12], and canines [13]. Substantial differences in sperm head shape and size were found within breeds in stallions [14,15], rams [16], bulls [17], alpacas [18], red deer [19], and boar [20]. Between-breed differences were identified in stallions [14], canines [21], bulls [17], boar [22], and buffalo [23]. Such variability could be, in part, due to genotypic effects [24].

There are two main horse groups in Thailand: purebred and Thai native crossbred horses. The country has a total population of 2,327 horses (Statistics of Livestock in Thailand: 2006, Department of Livestock Development, Ministry of Agriculture and Cooperatives). Purebred horse strains include Arabians, Standard-bred, Thoroughbred and Warm-blood, all of which were originally introduced to Thailand by importation. The Thai native crossbred horse is a pony horse that may have originated from a Burmese breed [25]. However, the scientific origin of the breed remains obscure. Nowadays, this native breed is generally used in religious ceremonies, for recreational activities, and occasionally for transportation in highland areas. Natural breeding with stallions is commonly performed to increase horse numbers. However, applications of reproductive technology in Thai native crossbred horses such as chilled semen or frozen semen are not available. Artificial insemination with chilled or frozen-thaw semen may be an important method for increasing population numbers of this horse, and a detailed study of its semen characteristics was therefore considered necessary.

The study aims to 1) investigate the sperm morphology and normal sperm head morphometry of Thai native crossbred animals, and 2) compare the obtained results with the characteristics of purebred stallion sperm of proven fertility.

## Materials and methods

### Chemicals

All chemicals in this study were purchased from Sigma Chemical Company (Sigma, St Louis, MO, USA) unless otherwise stated.

### Animals and Semen Collection

The investigation was performed on nine clinically healthy stallions, of which five were Thai native crossbreds (T: T1 – T5) and four were purebred animals of proven fertility (F: F1 was a Standard-bred; F2 was a Warm-blood; F3 and F4 were Thoroughbreds). All were aged between 5 and 12 years. Semen was collected using a Missouri-type artificial vagina on a monthly basis over the period January through June 2007 for a total of six ejaculates per stallion. Multiple semen parameters were routinely determined including volume, color, consistency, motility, progressive motility, and concentration. All the ejaculates were analyzed to evaluate sperm morphology. In the case of sperm head morphometry assessment, we used only the final four ejaculates in our analysis.

### Sperm Morphology Examination

Sperm morphology was studied in wet preparations comprising samples fixed in formal-saline [26] under a phase-contrast microscope (Olympus, Tokyo, Japan) at a magnification of 1000×. A total of 200 sperm in each ejaculate were examined for morphological abnormalities according to the criteria defined by Dowsett et al. [27]. Certain findings of abnormalities in the T sperm group were further examined under eosin/nigrosin staining or using scanning electron microscopy.

### Sperm Head Morphometry Measurements

A 200 μl semen sample was washed and diluted with Dulbecco's phosphate-buffered saline (DPBS) to a concentration of approximately 100 × 10^6 ^sperm/ml. Smears were prepared by taking a 7 μl drop of the diluted sperm, smearing it across a clean glass slide, and air-drying overnight.

#### Staining procedures

The sample slides were stained for 40 min with Harris' haematoxylin technique [28], and were permanently mounted before the sperm head was measured.

#### Head measurement

The slide was loaded into an IVOS version 12.3 microscopy system (Hamilton Thorne Research, Beverly, MA, USA) with the aid of a computer-controlled specimen stage. The images were evaluated using commercial morphology software (Oval Metrix Version 4.18). Recognition of sperm and the rejection of other cells were performed at an accuracy consistent with the hardware and software specifications. The analysis software settings were minimum contrast 15, minimum size 1 μm^2^, erosion level 7.0, camera gain 50, camera contrast 180, and scale 0.147 μm/px. The manufacturer-recommended objective magnification for equine sperm microscopy was 60×. 200 morphologically normal sperm heads were acquired in each test, and consequently a total of 800 sperm were analyzed for each animal. The software reported five sperm head features, namely length (L; μm), width (W; μm), elongation [(width/length) × 100; %], perimeter (P; μm) and head area (A; μm^2^). In addition, the software calculated four non-dimensional derived parameters, namely ellipticity (e) = (L - W)/(L + W); shape factor 1 (Sf1; rugosity) = 4πA/P^2^; shape factor 2 (Sf2) = Sf1 × (L/W) and shape factor 3 (Sf3; regularity) = π L/W/4A [18].

### Statistical Analyses

Statistical comparisons were made using the SPSS/PC+ statistics package (version 12.0 for Windows, SPSS Inc, Chicago, IL, USA). For each morphometric parameter, the normality and homogeneity of the data's variance distribution were assessed using the Kolmogorov-Smirnov and Levene's tests. One-way ANOVA producing significant F-values was followed by an LSD test for comparisons between multiple animals. An independent-samples T test was used for comparisons between groups of animals. All data given were summarized as mean ± standard error of the mean (SE). The coefficient of variation (CV) was calculated for both within-animal and between-animal groups [18].

## Results

The color and aspect of the ejaculates ranged from milky white to opalescent white. For T stallions, the mean ± SE of gel free-volume, motility, progressive motility, living sperm and concentration were 44.0 ± 2.1 ml, 77.8 ± 1.3%, 55.4 ± 1.3%, 75.5 ± 1.3%, 309.0 ± 30.7 × 10^6^sperm, respectively. For the F group, the mean ± SE of gel free-volume, motility, progressive motility, live sperm and concentration were 47.0 ± 3.2 ml, 73.0 ± 2.0%, 46.8 ± 1.7%, 73.9 ± 1.6%, 374.5 ± 28.4 × 10^6 ^sperm, respectively.

### Sperm Morphology

**Figure 1:**
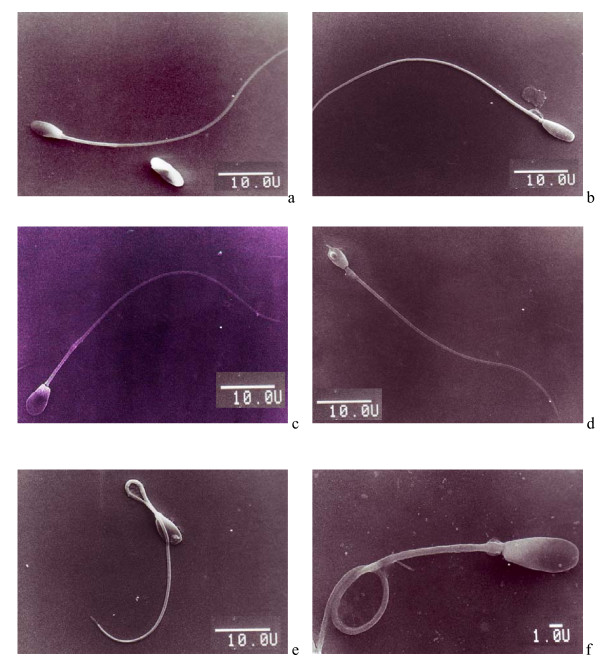
**Scanning electron microscopy of Thai native crossbreed stallion sperm;** a – normal sperm (top) and loose narrow head (below); b – narrow head with proximal cytoplasmic droplet; c – round head; d – acrosomal defect; e – acrosomal defect and bent tail and f – proximal cytoplasmic droplet with coiled tail (a-e – 2000×, bar = 10 micrometers; f – 3600x, bar = 1 micrometers).

**Figure 2:**
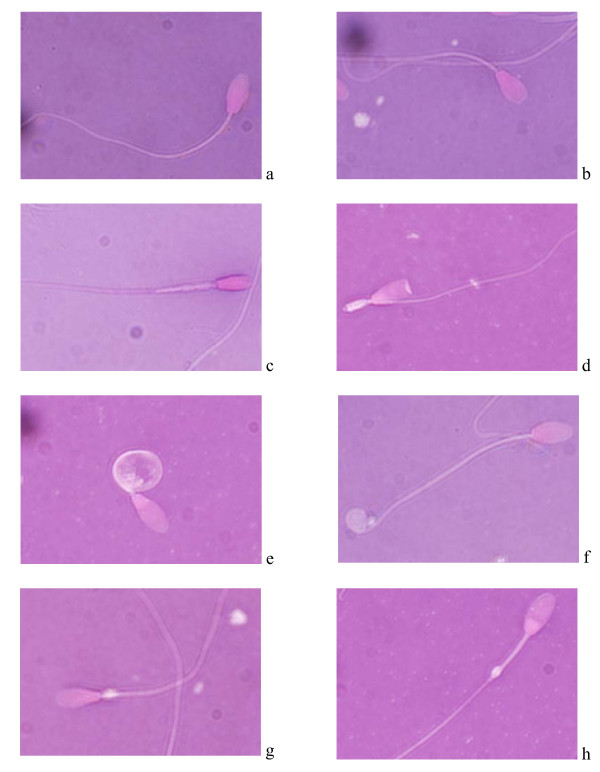
**Light microscopy of Thai native crossbreed stallion sperm after eosin/nigrosin staining;** a – normal sperm; b – pear shaped head; c – narrow head with abnormal midpiece; d – acrosomal defect with abnormal midpiece; e – coiled tail below head; f – terminal coiled tail; g – proximal cytoplasmic droplet and h – distal cytoplasmic droplet (1000×).

Morphology measurements from the individual ejaculate samples of T and F stallions are presented in Table 1. Sperm morphology varied among stallions with respect to all parameters. On average, the T and F groups were not significantly different (P > 0.05) in respect of percentage of sperm that exhibited normal morphology. However, the percentages of each type of morphologically abnormal sperm were significantly different (P < 0.05). Overall, the most common abnormality in both T and F stallions comprised sperm that had an abnormal midpiece. Morphologically normal and abnormal sperm from the T group are shown in Figure 1 (detected by scanning electron microscopy) and Figure 2 (stained with eosin/nigrosin and detected by light microscopy).

**Table 1:** Percentage of sperm morphology of Thai native crossbred (T; T1 – T5) and purebred (F; F1 – Standard-bred; F2 – Warm-blood; F3 and F4 – Thoroughbred) stallions (mean ± SE)

Stallion	Normal morphology	Abnormal head	Abnormal midpiece	Abnormal tail	Proximal cytoplasmic droplet	Distal cytoplasmic droplet
T1	48.9 ± 1.8 ^a^	8.9 ± 0.7 ^a^	12.7 ± 0.8 ^a^	1.1 ± 0.2 ^a^	10.9 ± 0.8 ^a^	17.6 ± 1.4 ^a^
T2	43.8 ± 2.4 ^a,c^	14.2 ± 1.5 ^b^	29.3 ± 1.6 ^b,c^	1.8 ± 0.4 ^a,b^	8.2 ± 1.1 ^a,b^	2.9 ± 0.5 ^b^
T3	60.8 ± 2.7 ^b^	11.5 ± 1.4 ^a,b^	16.2 ± 1.6 ^a,c^	1.4 ± 0.3 ^a^	4.9 ± 1.2 ^b,c^	5.2 ± 1.1 ^b,c^
T4	38.3 ± 2.7^c^	9.2 ± 1.1 ^a^	10.3 ± 0.8 ^a^	2.6 ± 0.5 ^b,c^	28.9 ± 2.2 ^d^	10.6 ± 1.7 ^d^
T5	58.3 ± 2.2 ^b^	9.1 ± 1.4 ^a^	19.8 ± 1.6 ^a^	1.6 ± 0.3 ^a,c^	3.3 ± 0.5 ^c^	7.9 ± 1.1 ^c,d^

Mean T (range)	49.7 ± 1.3 (19 – 75)	10.2 ± 0.5 * (2 – 33)	16.5 ± 0.8 * (3 – 41)	1.6 ± 0.1 * (0 – 7)	11.4 ± 1.0 * (0 – 42)	10.5 ± 0.8 * (0 – 36)

F1	40.1 ± 3.6 ^a^	13.8 ± 1.3 ^a^	31.6 ± 3.4 ^a,b^	5.4 ± 1.3 ^a^	3.5 ± 0.8 ^a^	5.5 ± 2.0
F2	61.5 ± 2.7 ^b^	10.2 ± 2.1 ^a^	14.3 ± 2.5 ^a^	2.7 ± 1.0 ^b^	5.7 ± 1.2 ^a,b^	5.5 ± 2.1
F3	35.9 ± 3.9 ^a^	17.3 ± 2.8 ^b^	29.9 ± 3.2 ^b^	0.3 ± 0.2 ^c^	9.3 ± 2.0 ^b^	7.4 ± 1.5
F4	58.0 ± 5.1 ^b^	11.7 ± 1.6 ^a^	17.3 ± 2.8 ^a^	4.4 ± 1.1 ^a,b^	3.9 ± 0.8 ^a,b^	4.7 ± 1.3

Mean F (range)	48.1 ± 2.8 (21 – 72)	13.4 ± 1.1 (4 – 23)	23.9 ± 2.1 (9 – 43)	3.3 ± 0.6 (0 – 13)	5.5 ± 0.7 (1 – 17)	5.8 ± 0.9 (0 – 16)

Significant differences (*P *< 0.05) within T or F are indicated by different letters (a, b, c, d).

Significant differences (*P *< 0.05) between T and F are indicated by * in superscript

The mean of numbers of morphologically normal sperm with progressive motility in each ejaculate ranged from 1.83 ± 0.37 to 4.68 ± 0.47 billion and 3.18 ± 0.53 to 5.41 ± 1.61 billion in T and F stallions, respectively.

### Sperm Head Morphometry

Parameters for the morphometric characteristics of normal sperm heads are summarized in Table 2. There were differences (P < 0.05) between individual stallions in both T and F. Comparisons between the mean values of each characteristic of normal T and F sperm heads showed that the length, elongation, perimeter and area values were higher for T than for F (P < 0.05). Thus, this indicated that the sperm heads of T stallions were rounder and larger than those of F stallions were.

**Table 2:** Normal sperm head morphometry of Thai native crossbred (T; T1 – T5) and purebred (F; F1 – Standardbred; F2 – Warmblood; F3 and F4 – Thoroughbred) stallions (mean ± SE)

Stallion	Length (μm)	Width (μm)	Elongation (%)	Perimeter (μm)	Area (μm^2^)	Ellipticity	Sf1	Sf2	Sf3
T1	6.24 ± 0.03^a^	2.99 ± 0.02^a^	0.35 ± 0.002^a^	15.98 ± 0.04^a^	15.88 ± 0.09^a,b^	2.09 ± 0.01^a^	0.78 ± 0.002^a^	1.63 ± 0.006^a^	0.92 ± 0.004^a^
T2	6.07 ± 0.02^b^	3.19 ± 0.02^b^	0.31 ± 0.003^b^	15.73 ± 0.04^b^	16.23 ± 0.09^b^	1.90 ± 0.01^b^	0.82 ± 0.002^b^	1.57 ± 0.007^b^	0.94 ± 0.003^b^
T3	6.33 ± 0.02^c^	3.19 ± 0.01^b^	0.33 ± 0.002^c^	16.10 ± 0.04^a^	16.67 ± 0.08^c^	1.98 ± 0.01^c^	0.81 ± 0.001^c^	1.60 ± 0.005^c^	0.95 ± 0.002^b^
T4	6.18 ± 0.02^ab^	3.11 ± 0.02^c^	0.33 ± 0.003^c^	15.76 ± 0.04^b^	15.90 ± 0.09^a,b^	1.99 ± 0.01^c^	0.80 ± 0.002^c^	1.60 ± 0.008^b,c^	0.95 ± 0.004^b^
T5	6.18 ± 0.02^a^	3.08 ± 0.01^c^	0.33 ± 0.002c	15.71 ± 0.03^b^	15.77 ± 0.07^a^	2.02 ± 0.01^c^	0.80 ± 0.002^c^	1.61 ± 0.005^a,c^	0.95 ± 0.002^b^

Mean T	6.22 ± 0.01*	3.09 ± 0.0*	0.34 ± 0.001*	15.88 ± 0.02*	16.09 ± 0.03*	2.01 ± 0.005*	0.80 ± 0.001*	1.61 ± 0.003*	0.94 ± 0.002*
% CV (T)	4.5	6.2	8.5	3.3	6.6	6.5	3.3	4.6	4.2
Percentile 25/75	6.00/6.40	2.90/3.20	0.31/0.35	15.60/16.20	15.40/16.80	1.94/2.10	0.79/0.82	1.56/1.66	0.92/0.97

F1	5.94 ± 0.02^a^	2.99 ± 0.01^a^	0.33 ± 0.002^a^	15.14 ± 0.03^a^	14.83 ± 0.07^a^	1.99 ± 0.07^a^	0.81 ± 0.001^a^	1.62 ± 0.004^a^	0.94 ± 0.002^a^
F2	5.98 ± 0.01^a^	2.92 ± 0.01^b^	0.34 ± 0.002^b^	14.89 ± 0.03^b^	13.97 ± 0.05^b^	2.06 ± 0.01^b^	0.79 ± 0.002^b^	1.62 ± 0.004^a^	0.98 ± 0.001^b^
F3	6.15 ± 0.03^b^	2.92 ± 0.01^b^	0.36 ± 0.002^c^	15.24 ± 0.05^a^	14.41 ± 0.09^c^	2.11 ± 0.01^c^	0.78 ± 0.002^c^	1.64 ± 0.006^b^	0.98 ± 0.003^bc^
F4	5.85 ± 0.01^c^	2.80 ± 0.01^c^	0.35 ± 0.001^c^	14.55 ± 0.03^c^	13.20 ± 0.05^d^	2.09 ± 0.01^c^	0.79 ± 0.001^c^	1.64 ± 0.004^b^	0.98 ± 0.002^c^

Mean F	5.94 ± 0.08	2.89 ± 0.01	0.35 ± 0.001	14.88 ± 0.02	13.90 ± 0.04	2.06 ± 0.004	0.79 ± 0.001	1.63 ± 0.002	0.97 ± 0.001
% CV (F)	5	6.5	8.8	4	8.5	6.8	3.4	4.7	3.5
Percentile 25/75	5.70/6.20	2.80/3.10	0.33/0.36	14.50/15.20	13.10/14.70	1.97/2.14	0.77/0.81	1.58/1.68	0.95/0.99

Significant differences (P < 0.05) within T or F are indicated by different letters (a, b, c, d).

Significant differences (P < 0.05) between T and F are indicated by * in superscript

The percentage CV values of normal sperm head morphometric characteristics were quite low, ranging from 3.3 (shape factor 1 and perimeter) to 8.5 (elongation) and 3.4 (shape factor 1) to 8.8 (elongation) in T and F stallions, respectively (Table 2). Within-stallion group analysis indicated that the CV values in both T and F sperm were also low (Table 3), while analysis of the between-animal groups found that the percentage CVs were quite high for perimeter (19.2), area (19.8), length (28.9), shape factor 1 (25.8) and shape factor 3 (32.0) for T stallions, but were only high in the case of shape factor 1 (26.1) for F sperm. The sperm head parameters with a low within-animal CV and a high between-animal CV were perimeter (2.9, 19.1), shape factor 1 (2.6, 25.8) and shape factor 3 (3.8, 32.0) for T. The latter characteristics were observed only in the shape factor 1 variable (2.9, 26.1) for F sperm.

**Table 3:** Within-animal and between-animal CV of normal sperm head morphometry in Thai native crossbred (T) and purebred (F) stallions

Stallion	Length	Width	Elongation	Perimeter	Area	Ellipticity	Sf1	Sf2	Sf3
T									
Within-animal CV	4.0	5.4	7.5	2.9	5.9	5.6	2.6	4.3	3.8
Between-animal CV	28.9	17.6	11.2	19.2	19.8	13.9	25.8	13.2	32.0

F									
Within-animal CV	4.8	5.8	8.1	3.6	7.2	6.3	2.9	4.5	3.2
Between-animal CV	5.3	11.9	17.6	1.6	4.0	16.0	26.1	14.2	7.2

## Discussion

The percentages of each type of sperm morphology were variable across both T stallions and F stallions. Inter-animal variation was found both within breeds [29] and between breeds [29-31]. The overall percentage of morphologically normal sperm was 49.7% and 48.1% for T and F stallions, respectively, which closely matches the 50% value that is considered a "normal" average for stallions [8]. Our finding is consistent with the 43.4% morphologically normal sperm with acceptable fertility [6], but lower than the value for fertile stallions (75.5%) reported by Pesch et al. [7]. For morphologically abnormal sperm, high numbers of sperm presented with an abnormal midpiece in both T (16.5%) and F stallions (23.9%). A high proportion of sperm with midpiece abnormalities (25.3%) has also been reported by Voss et al. [32]. However, in this study, the stallions nonetheless achieved acceptable pregnancy rates of 62.5 to 91.7% [32]. One reason why the sperm specimens may have had abnormal midpiece morphology may have been due to a response to environmental insults as seen in bull scrotal insulation studies [33,34]. In addition to impaired epididymal function, insults to spermatocytes or spermatids are also known to result in an increase in cytoplasmic droplet concentration in bull sperm samples [33]. Our study found higher percentages of both proximal and distal cytoplasmic droplets in T stallions than in F stallions. However, these types of abnormality may [7,35] or may not [32,36] affect stallion fertility. A greater impact of sperm abnormality on fertility could be caused by an abnormal head, especially a detached acrosome, as well as by a breakdown in the structural integrity of the plasma membrane and other important organelles. The latter could be identified under transmission electron microscopy [7,37].

All stallions had more than one billion morphologically normal, progressively motile sperm per ejaculate. On this basis, it might be assumed that all the T stallions were fertile, and that they were of comparable fertility to the proven-fertile F stallions. However, their actual fertility or pregnancy rate was not tested in this study.

The morphometric characters of normal sperm heads were significantly different among individual T or F stallions, and between T and F stallions. Differences in sperm head size within breed have been reported in both Warm-blood [14] and Spanish thoroughbred stallions [15]. Similarly, differences between breeds have been observed in Arabian, Warm-blood, Thoroughbred and Morgan stallions [14]. The results of this study confirm that there is significant variation in normal sperm head characteristics both within and between various breeds of stallions, including the Thai native crossbred. In general, sperm in the T group were larger and rounder than those in the F group were. This may render T sperm more sensitive to certain types of extenders that are commonly employed in cooled storage semen [38]. The cooling rate for stallion sperm can affect sperm motility during storage [39,40]. Sperm of different sizes may undergo different cooling rates during a single procedure. Other researchers have also found that the 'smaller' and 'more elongated' the sperm specimen, the better the sperm's cryoresistance [12]. Thus, sperm head size or shape may be an aspect to consider as part of efforts to improve cooled storage and cryopreservation protocols.

Compared to previous studies in which the Harris' hematoxylin technique was also used, almost all the morphometric parameters of F sperm heads in this study were higher than those of both sub-fertile stallions of unclassified breeds [9] and Spanish Thoroughbred stallions [28]. The values showing this property were, respectively, length (5.94 μm, 5.77 μm, 5.67 μm); width (2.89 μm, 2.89 μm, 2.85 μm); perimeter (14.88 μm, 14.59 μm, 15.00 μm) and area (13.90 μm^2^, 12.66 μm^2^, 13.42 μm^2^). Nevertheless, some parameters in our study were lower than those for certain unclassified breeds of stallions in a different research trial [41], which reported values as follows: length 6.01 μm, width 2.97 μm, perimeter 15.64 μm and area 13.48 μm^2^.

Within-animal group percentage CVs for all head morphometric parameters were low for sperm in both the T group (from 2.6 for shape factor 1 to 7.5 for elongation) and in the F group (from 2.9 for shape factor 1 to 8.1 for elongation). This reflected a homogeneous sperm population within individuals. These results were consistent with those studies which examined unclassified breeds of stallion (from 5.8 for length and perimeter to 8.8 for area) [41], ram (from 4.36 for length to 7.33 for shape factor 1) [16], boar (from 2.93 for rugosity or shape factor 1 to 9.38 for elongation) [20], but lower than those of the Cynomolgus monkey (from 2.90 for shape factor 1 to 16.39 for ellipticity) [42], or alpaca (from 4.7 for shape factor 1 to 17.8 for ellipticity) [18].

Between-animal group percentage CVs were higher in the sperm of T group animals (from 11.2 for elongation to 32.0 for shape factor 3) than in F group stallions (from 1.6 for perimeter to 26.1 for shape factor 1). Identification of individual animals might be possible if one focuses on those parameters that have low within-animal and high between-animal CVs. The literature suggest that suitable parameters for other species might include perimeter (5.42 versus 35.45) and shape factor 1 (7.33 versus 36.98) for rams [16], and perimeter (2.69 versus 14.43), shape factor 1 (rugosity; 2.93 versus 26.26) and shape factor 3 (regularity; 2.45 versus 16.31) for boars [20]. Meanwhile, our study suggested that perimeter (2.9 versus 19.2), shape factor 1 (2.6 versus 25.8) and shape factor 3 (3.8 versus 32.0) for T and shape factor 1 (2.9 versus 26.1) for F sperm were suitable parameters. The crossbred genetic background may result in increased between-animal sperm dimensional variability as compared with purebred groups.

## Conclusion

The results presented here indicate that the variability in percentages of normal and abnormal morphological characteristics of sperm in individual Thai native crossbred stallions was similar to that of purebred stallions. Furthermore, the morphometric characteristics of normal sperm heads also varied substantially between stallions, with the sperm heads of Thai native crossbred stallions being larger and rounder than those of purebred stallions. Perimeter, shape factor 1 and shape factor 3 were identified as parameters that could potentially be used as a means of identifying individual T stallions.

## Competing interests

The authors declare that they have no competing interests.
